# Therapeutic Exploitation of Neuroendocrine Transdifferentiation Drivers in Prostate Cancer

**DOI:** 10.3390/cells13231999

**Published:** 2024-12-03

**Authors:** Zoe R. Maylin, Christopher Smith, Adam Classen, Mohammad Asim, Hardev Pandha, Yuzhuo Wang

**Affiliations:** 1 Vancouver Prostate Centre, Department of Urological Sciences, University of British Columbia, Vancouver, BC V6H 3Z6, Canada; aclassen@bccrc.ca (A.C.); ywang@bccrc.ca (Y.W.); 2 Department of Experimental Therapeutics, BC Cancer Agency, Vancouver, BC V5Z 4E6, Canada; 3 Targeted Cancer Therapy, Department of Clinical and Experimental Medicine, Faculty of Health and Medical Sciences, University of Surrey, Guildford GU2 7WG, UK; m.asim@surrey.ac.uk (M.A.); h.pandha@surrey.ac.uk (H.P.)

**Keywords:** neuroendocrine, prostate, cancer, transdifferentiation, lineage plasticity

## Abstract

Neuroendocrine prostate cancer (NEPC), an aggressive and lethal subtype of prostate cancer (PCa), often arises as a resistance mechanism in patients undergoing hormone therapy for prostate adenocarcinoma. NEPC is associated with a significantly poor prognosis and shorter overall survival compared to conventional prostate adenocarcinoma due to its aggressive nature and limited response to standard of care therapies. This transdifferentiation, or lineage reprogramming, to NEPC is characterised by the loss of androgen receptor (AR) and prostate-specific antigen (PSA) expression, and the upregulation of neuroendocrine (NE) biomarkers such as neuron-specific enolase (NSE), chromogranin-A (CHGA), synaptophysin (SYP), and neural cell adhesion molecule 1 (NCAM1/CD56), which are critical for NEPC diagnosis. The loss of AR expression culminates in resistance to standard of care PCa therapies, such as androgen-deprivation therapy (ADT) which target the AR signalling axis. This review explores the drivers of NE transdifferentiation. Key genetic alterations, including those in the tumour suppressor genes RB1, TP53, and PTEN, and changes in epigenetic regulators, particularly involving EZH2 and cell-fate-determining transcription factors (TFs) such as SOX2, play significant roles in promoting NE transdifferentiation and facilitate the lineage switch from prostate adenocarcinoma to NEPC. The recent identification of several other key novel drivers of NE transdifferentiation, including MYCN, ASCL1, BRN2, ONECUT2, and FOXA2, further elucidates the complex regulatory networks and pathways involved in this process. We suggest that, given the multifactorial nature of NEPC, novel therapeutic strategies that combine multiple modalities are essential to overcome therapeutic resistance and improve patient outcomes.

## 1. Introduction

Prostate cancer (PCa) is the most frequently diagnosed cancer and ranks as the second leading cause of cancer deaths in men across the United States, and is the second most common cancer in men globally [1,2]. Although treatment has improved significantly, the prognosis for advanced PCa is poor, and many patients continue to succumb to the disease [2]. PCa is driven by androgens such as dihydrotestosterone (DHT) and their interaction with the androgen receptor (AR), a ligand-activated transcription factor (TF) that fuels growth, proliferation, and disease progression [3]. The standard first-line treatment for metastatic PCa typically involves androgen deprivation therapy (ADT) alongside other potent drugs that target the AR signalling axis, such as abiraterone acetate or enzalutamide [3]. Although these treatments often yield significant and lasting responses, resistance to AR-targeted therapies eventually develops. These cases are classified as castration-resistant prostate cancer (CRPC) and they continue to depend on AR signalling, which may persist due to mutations, amplifications, or other mechanisms that reactivate AR signalling [4]. This ongoing challenge has prompted the exploration of alternative strategies to further inhibit AR signalling.

Research has indicated that throughout the course of treatment, a significant percentage of CRPC tumours will no longer depend on AR signalling and will take on a wholly distinct histological appearance. Studies have linked the functional impact and consequence of AR pathway therapeutic inhibition and the acquisition of a neuroendocrine (NE) phenotype and subsequent lineage reprogramming to a clinical entity known as treatment-emergent neuroendocrine prostate cancer (t-NEPC) [5,6]. NEPC has been reported to be present in around 17% of patients with progressive, metastatic CRPC (mCRPC) following the biopsy of metastases [7]. Indeed, rapid autopsy studies have confirmed this finding by reporting a prevalence of 10–20% in mCRPC patients [8]. A diagnosis of NEPC is associated with poor clinical outcomes, and the median overall survival has been reported at less than one year following diagnosis [9]. Interestingly, NEPC may arise de novo; however, this is a rare clinical entity, accounting for less than 1–2% of all PCa cases at the initial diagnosis. The vast majority of cases of NEPC are thought to be clonally derived from prostate adenocarcinoma and are therefore diagnosed in patients previously treated with hormone therapy and AR pathway inhibitors [5,10,11,12,13]. Genomic analyses of human prostate biopsy and surgically excised samples support this model. For instance, the well-characterised TMPRSS2:ERG gene fusion was found to occur in approximately 50% of NEPC cases, a frequency comparable to that observed in prostate adenocarcinoma [14,15]. Further, early research revealed that prostate adenocarcinoma cells cultured in an androgen-depleted medium downregulate the expression of AR and its canonical target gene, PSA, and simultaneously acquire an NE phenotype [16]. This phenotype includes altered morphology with neurite-like processes and the presence of numerous cytoplasmic secretory granules, characteristic of NE cells [16]. This finding provided an early indication that castration in the form of ADT may be actively promoting the NE transdifferentiation process as a mechanism of resistance. Similarly, the prolonged exposure of adenocarcinoma patient-derived xenografts (PDXs) to ADT has been shown to promote CRPC and eventually a complete lineage switch to NEPC [17].

**Figure 1:**
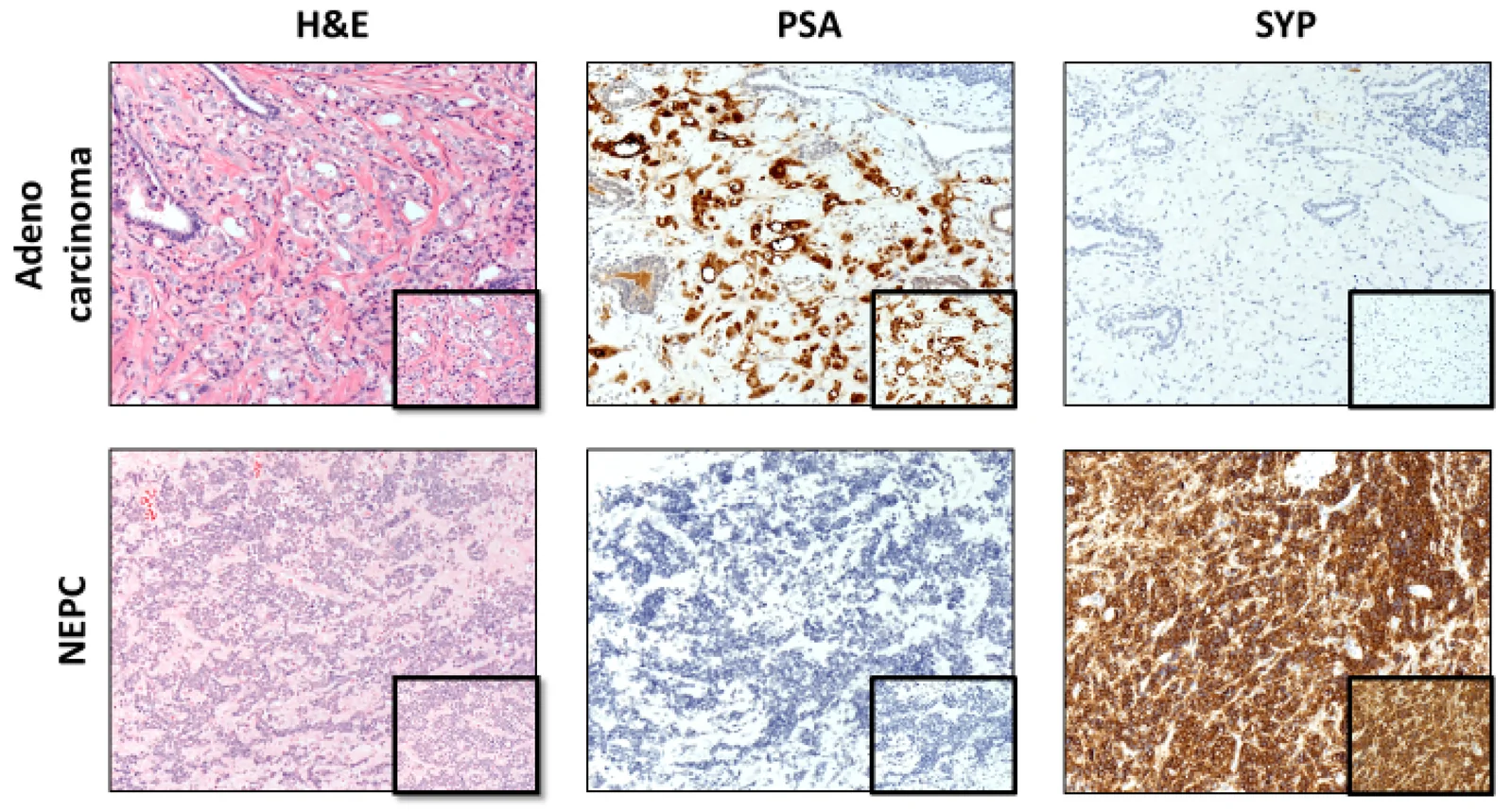
Immunohistochemical (IHC) analysis of prostate adenocarcinoma and neuroendocrine prostate cancer (NEPC). Representative sections from a patient with prostate adenocarcinoma and a patient with NEPC were stained for haematoxylin and eosin (H&E) to assess histology, prostate-specific antigen (PSA) to detect luminal epithelial differentiation, and synaptophysin (SYP) to indicate neuroendocrine differentiation. Prostate adenocarcinoma tissue displays high PSA expression and an absence of SYP staining, characteristic of luminal epithelial differentiation. In contrast, NEPC tissue exhibits strong SYP expression with minimal or absent PSA staining, reflecting neuroendocrine features. Images taken at 20× magnification, with 40× magnification in the corner. Patient tumours were collected as donor tissues for the patient-derived xenograft study [23].

**Figure 2:**
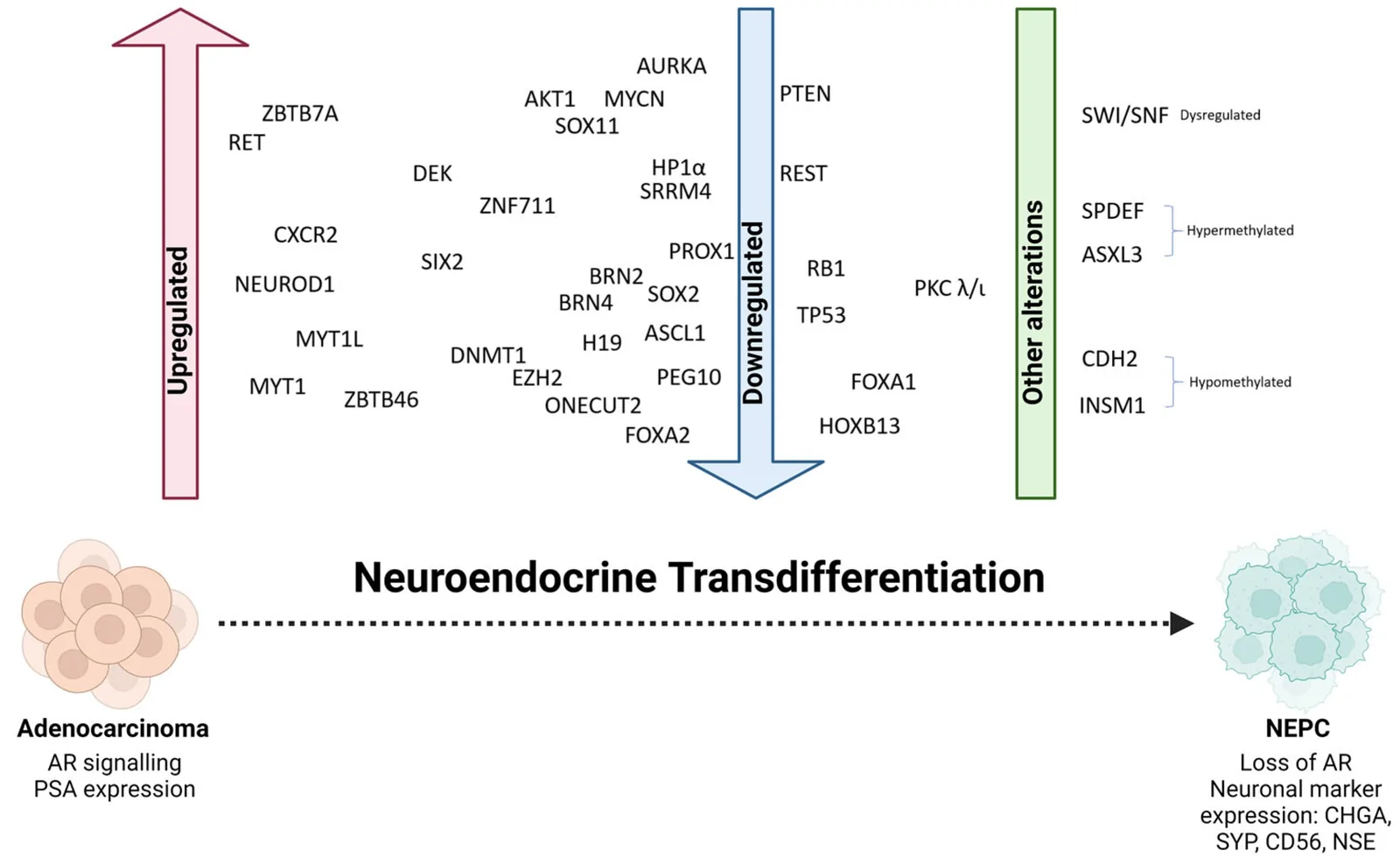
Key players in the neuroendocrine transdifferentiation process of prostate adenocarcinoma to neuroendocrine prostate cancer. Driving factors identified in the literature to facilitate the lineage plasticity and phenotypic reprogramming of prostate cancer cells, namely by the loss or downregulation of tumour suppressor genes, differentially expressed genomic and epigenetic drivers, dysregulated AR interactors and aberrations to other modulatory factors. Figure shows drivers altered state during the transdifferentiation process is upregulated/overexpressed, downregulated/loss, dysregulated, hypomethylated, hypermethylated.

NEPC is a highly proliferative disease that is negative for AR and PSA expression (Figure 1). The presence of NEPC can be suspected if there is an increase in disease burden without a concomitant rise in PSA and the emergence of metastatic deposits in visceral organs such as the lungs or liver [11]. Four core markers have been identified and are used on histology to identify NEPC in clinical samples: neuron-specific enolase (NSE), chromogranin-A (CHGA), synaptophysin (SYP), and neural cell adhesion molecule 1 (NCAM1/CD56) [10,18,19]. Other NE markers have been discovered; however, these four are the common markers that researchers and pathologists have used consistently [18,19,20]. The treatment of NEPC remains a significant clinical challenge due to its aggressive nature and resistance to conventional therapies targeting the androgen receptor (AR) signalling axis. Chemotherapy regimens, including platinum-based agents like cisplatin and etoposide, have been used with some success, reflecting treatment protocols for other neuroendocrine tumours, such as small-cell lung cancer (SCLC) [21]. NEPC generally exhibits initial sensitivity to platinum-based chemotherapy, with the objective response rates ranging from 50% to 60% [22]. While platinum-based chemotherapy can provide an initial response in some NEPC patients, the median response duration of 5.8 months, and the treatment regimen comes with a range of potential toxicities such as severe neutropenia [10]. This emphasises the need for novel targeted treatment strategies through ongoing molecular and clinical research. This review focuses on the driver landscape of t-NEPC, including tumour suppressor gene (TSG) alterations, epigenetic factors, genomic drivers, alterations in AR interactors, and other modulatory factors (summarised in Figure 2). From this point forward, t-NEPC and NEPC will be used interchangeably. Unveiling, the mechanistic underpinnings of the NE transdifferentiation process may enable the ability to estimate the risk of transdifferentiation to NEPC in patients with CRPC, and combat treatment resistance by highlighting novel druggable targets. The resistance of NEPC to standard-of-care treatment makes this variant hard to treat, and currently, there are no clinically available targeted therapies. Therefore, there is an urgent need to gain a more comprehensive understanding of this lethal variant, for example, in terms of the genetic landscape, and thus create more personalised and targeted therapies to improve the patient quality of life and longevity.

## 2. Genetic and Epigenetic Drivers of Neuroendocrine Prostate Cancer

### 2.1. Tumour Suppressor Alterations

Tumour suppressor genes (TSGs) are genes that play a crucial role in regulating cell growth and division, helping prevent the development of cancer, often encoding for proteins involved in the cell cycle, DNA damage repair, apoptosis, and proliferation [24]. Understanding the nature of TSG alterations in NEPC is important for understanding this aggressive variant, developing targeted therapies, combatting treatment resistance, and improving diagnostic and prognostic capabilities. A comprehensive understanding can ultimately lead to better patient management and outcomes.

Genomic alterations, such as mutations and deletions in the TSGs RB transcriptional corepressor 1 (RB1, encoding for Rb) and tumour protein P53 (TP53, encoding for p53) are commonly observed in other NE tumour types, such as SCLC [25]. Clinically, PCa tumours exhibiting combined RB1 and TP53 loss showed increased resistance to a broad spectrum of therapeutics and vulnerability to replication stress. They also demonstrated greater proliferation rates and elevated DNA repair activity [26]. The recent molecular characterisation of NEPC has revealed that alterations in these genes and the TSG phosphatase and tensin homolog (PTEN) are enriched in NEPC compared with prostate adenocarcinoma. One study found retinoblastoma protein (Rb) loss in 90% and RB1 allelic loss in 85% of NEPC cases [27]. In the same study, p53 protein loss was observed in 56% of NEPC cases, with 60% showing TP53 mutation. The loss of PTEN protein was detected in 63% of NEPC cases, with 38% having allelic loss. A later study showed similar findings, concluding that combined alterations in RB1, TP53, and/or PTEN were more frequent in NEPC than in CRPC, and that the RB1 copy number loss was the strongest discriminator between the two groups [28].

The functional relationship between the alterations in the Rb and p53 pathways has been identified in NEPC development. Several studies have described how these pathways cooperate to suppress PCa lineage plasticity and antiandrogen resistance. A recent study demonstrated that the in vitro knockout of both genes altered the expression of over 1000 genes and resulted in a reduction in the AR transcriptional programme without any alterations in the AR [26]. Another study used an in vitro dual-knockdown approach of both TSGs, and demonstrated the promotion of lineage plasticity and enzalutamide resistance, mediated by an increased expression of SRY-related HMG-box 2 (SOX2) [29]. A further finding corroborated this in an in vivo mouse model, highlighting that RB1 loss promotes the lineage plasticity and metastasis of prostate adenocarcinoma, which is initiated by PTEN mutation [29]. Additionally, the loss of TP53 confers resistance to antiandrogen therapy [30]. The authors concluded that RB1 and TP53 are necessary to repress the expression of epigenetic reprogramming factors, such as SOX2 and Enhancer of Zeste 2 Polycomb Repressive Complex 2 Subunit (EZH2), and that the loss of these TSGs can foster a stem cell-like epigenetic environment, promoting lineage plasticity, progression to NEPC, and treatment resistance.

The restoration of these TSGs could potentially suppress or even reverse tumorigenesis in NEPC. A type of gene therapy that reintroduces wild-type p53 using an adenoviral vector, often in the form of a vaccine, has been tested in preclinical studies and clinical trials for a wide array of malignancies [31,32,33], including PCa [34,35]. These studies have shown promising results, but positive outcomes depend on the p53 status of the patient; therefore, making this treatment more specific for NEPC patients would suggest more efficacious results. Comparable approaches have been taken for Rb loss; however, this has been studied much less extensively. In vitro and in vivo results for the treatment of retinoblastoma determined that gene therapy reintroducing the Rb protein was an effective strategy at treating the disease [36]. With just 10% of NEPC cases maintaining Rb protein expression, this treatment avenue could offer significant therapeutic potential. Finally, PTEN reintroduction by similar methods as above has proven effective in preclinical studies in a variety of cancer models [37,38,39,40].

Other mitigation strategies to alleviate the effects of these TSG losses in cancers have also been trialled. For example, a phase III trial (NCT04493853) in hormone-sensitive PCa with PTEN deficiency demonstrated the combination treatment of abiraterone with an inhibitor of AKT (which is a downstream target of PTEN); capivasertib is currently ongoing [41]. This trial was a consequence of the successful results of another trial (NCT03072238), demonstrating prolonged radiographic progression-free survival with a similar treatment regimen [42]. Additionally, a breast cancer study demonstrated that exploiting Rb loss by targeting cell cycle checkpoint proteins offers a palpable therapeutic option [43]. Indeed, PARP inhibitors have shown great promise in patients with a co-deletion of RB1 and BRCA2 in PCa with one inhibitor, Olaparib, obtaining FDA approval [44,45,46]. Furthermore, in cancers with downregulated wild-type p53, a few phase I clinical trials are being completed to target the negative regulators of p53, MDM2/MDMX (NCT01636479, NCT01985191, NCT01877382) [47]. One drug, Milademetan, is currently being repurposed since its FDA approval for Li–Fraumeni Syndrome. These mitigation efforts discussed are a small sample of the large strategy to target the effects of the loss of TSGs in cancer. Therefore, there are many therapeutic avenues that should be explored here for the treatment of NEPC which might be made more effective in combination with other treatments.

### 2.2. Epigenetic Factors

Epigenetics is the study of heritable changes in gene function that occur without alterations to the DNA sequence itself. This often entails DNA modifications (methylation), histone modifications (acetylation, methylation, SUMOylation, phosphorylation, ubiquitination), or the activity of non-coding RNAs [48]. Investigating epigenetic gene alterations in NEPC is crucial for understanding the mechanisms underlying NE transdifferentiation and lineage reprogramming. Furthermore, a better understanding of the epigenetic basis of NEPC will aid in identifying diagnostic and prognostic biomarkers, developing targeted and combination therapies, overcoming treatment resistance, and advancing personalised medicine.

As mentioned, Rb and p53 loss has been shown to mediate the upregulation of SOX2 that contributes to lineage plasticity and NE transdifferentiation. Furthermore, the LIN28B/Let-7 signalling axis has also been demonstrated to be activated to induce SOX2 expression in NEPC [49]. SOX2 can repress typical prostate adenocarcinoma genes due to its activation of lysine-specific demethylase 1 (LSD1) that causes the marked global hypomethylation of histone H3 [50]. These SOX2-mediated epigenetic changes downregulate AR and its associated target genes, enabling an increase in NE gene expression. While there are no direct inhibitors of SOX2, targeting upstream regulators or downstream targets could be a tenable option. There are a few competitive inhibitors of LIN28 that block the binding of miRNA Let-7 that could be repurposed for NEPC treatment. All potential therapies seem to be in the early phases of development; however, therapeutics such as TPEN and LI71 are showing promise [51]. The EGFR signalling pathway is known to be upstream of SOX2, and thus inhibitors such as gefitinib, erlotinib, and dasatinib could reduce the impact of Rb and p53 loss downstream [52]. Targeting LSD1 as SOX2’s prominent downstream target in NEPC could also be a tenable option. Many LSD1 inhibitors are in clinical trials for the treatment of various cancers, particularly SCLC and acute myeloid leukaemia (AML). Therapeutics such as TCP, ORY-1001, GSK-2879552, and IMG-7289 [53] have all demonstrated LSD1 inhibition and could be repurposed for the treatment of NEPC.

The EZH2 protein forms the catalytic component of the polycomb repressive complex 2 (PRC2), and its major function involves the trimethylation of histone 3 at lysine residue 27 (H3K27me3), resulting in the local formation of heterochromatin and transcriptional repression [54]. EZH2 has been shown to be crucial for the continued proliferation of in vitro cancer cell lines, and the ectopic expression of this protein can confer a proliferative advantage in primary cells [55]. Indeed, mutation and overexpression have been observed in many cancers, including NEPC [56]. Hormone-naive PDX adenocarcinoma model, LTL331, and castration-resistant NEPC subline, LTL331R, reflect this finding with the upregulation of EZH2 expression in the latter [54]. Furthermore, it has been shown that ADT can promote NE transdifferentiation and angiogenesis through EZH2 [57,58]. The Beltran group recently published a study confirming that in NEPC, EZH2 has non-canonical functions compared to its canonical roles in prostate adenocarcinoma. In NEPC, EZH2 upregulates NEPC-associated transcriptional drivers and neuronal gene programs, and contributes to a more aggressive, stem-like phenotype. Furthermore, the EZH2-mediated regulation of cell cycle genes are lost in NEPC, unlike in adenocarcinoma [59], suggesting increased cell cycle dysfunction.

Mutations in EZH2 found across various cancer types have prompted efforts to target it therapeutically in cancers characterised by epigenetic dysregulation. Given that EZH2 functions as an oncogenic driver and is overexpressed in several malignancies, such as NEPC, specific small-molecule inhibitors designed to block its methyl transferase activity are currently advancing through the pre-clinical and clinical development stages. [56]. The earliest of these inhibitors, 3-deazaneplanocin A (DZNep), was tested in AR-negative NEPC cell lines, and the inhibition of EZH2 resulted in AR re-expression and growth inhibition, implying that this group of drugs has the potential to reverse the NE transdifferentiation process by lifting epigenetic silencing mechanisms [60]. The authors concluded that this result can only be attributed in part to EZH2 inhibition, as recent findings have revealed that DZNep functions as a broad-spectrum histone methylation inhibitor, indicating that its effects are not specific to EZH2 [61]. Tazemetostat, a more potent and orally available EZH2 inhibitor, has recently been approved by the FDA for the treatment of advanced epithelioid sarcoma (NCT02601950). Furthermore, it has demonstrated a favourable safety profile and antitumour activity in a phase I clinical trial for B-cell lymphoma and advanced solid tumours [62], and is currently undergoing phase II studies (NCT02875548). In line with this, tazemetostat has recently entered a phase I clinical trial for the treatment of chemotherapy-naïve, progressive mCRPC patients in combination with enzalutamide or abiraterone/prednisone against these treatments alone (NCT04179864). Importantly, the trial inclusion criteria include histologically confirmed small cell or NE tumours of the prostate. Mirzaei et al. also proposed that non-coding RNAs directly or indirectly modulate EZH2 activity. Several micro-RNAs such as miR-101 and miR-26a, and long noncoding RNAs (e.g., MALAT1 and TUG1) have demonstrated regulation of EZH2 in multiple cancers and can also have potential as therapeutic targets [63].

Another interesting epigenetic mechanism facilitating the NE transdifferentiation process in PCa involves heterochromatin protein 1α (HP1α) (encoded by CBX5). HP1α is a highly conserved nonhistone protein involved in the formation of functional kinetochores that connect chromosomes to microtubules during mitosis, and is also associated with heterochromatin and gene silencing [64]. This role of this protein in NEPC was discovered using a combination of Living Tumor Lab (LTL) PDX models, patient cohorts, and genetically engineered mouse models (GEMMs), which demonstrated an enrichment of HP1α in NEPC. Most importantly, the researchers were able to perform a longitudinal analysis using their LTL331/331R model, harvesting samples in the weeks following the castration of LTL331-bearing mice and, importantly, throughout the NE transdifferentiation process. HP1α expression was shown to increase as an early event and rise steadily during transdifferentiation and lineage reprogramming to NEPC, and remained highly expressed in the tumour [65]. Interestingly, knocking down HP1α caused the inhibition of proliferation, colony formation, and induced apoptosis in NCI-H660 NEPC cells. Furthermore, HP1α ectopic expression in adenocarcinoma cells subjected to ADT promoted NE transdifferentiation in a mechanism that involves transcriptional repression on the promotors of AR and REST. At present, no small molecule inhibitors of HP1α have been developed; however, this protein remains to be an interesting target for NEPC treatment and should be explored further. Moreover, HP1α interacts with and is phosphorylated by the NDR (Nuclear Dbf2-Related) family of kinases during mitosis, regulating its localisation and function in chromosome alignment [66]. The targeting of kinases in cancer therapies has previously demonstrated considerable efficacy; therefore, this may be an easier option for HP1α inhibition. However, there are also no known inhibitors of this family of proteins at this current stage.

Other distinct epigenetic changes in NEPC have been documented such as dysregulation of the SWItch/sucrose non-fermentable (SWI/SNF) chromatin remodelling complex, RB1 loss or TP53 loss-driven upregulation of DNA methyltransferase 1 (DNMT1), hypermethylation of SAM pointed domain-containing Ets transcription factor (SPDEF) and ASXL Transcriptional Regulator 3 (ASXL3) and hypomethylation of Insulinoma-associated 1 (INSM1) and Cadherin 2 (CDH2). These epigenetic changes have shown to contribute to the NE phenotype and have potential as markers for the non-invasive detection of NEPC and as therapeutic targets [67,68,69].

### 2.3. Genomic Neuroendocrine Drivers

The expression of protein-coding genes, particularly transcription factors (TFs) and their associated proteins, can change with the development and progression of cancer, with significant influence on the activity and characteristics of the cancer [70]. Exploring and characterising these genomic changes in NEPC is paramount for a comprehensive understanding of the drivers of NE transdifferentiation, identifying new therapeutic targets and diagnostic and prognostic biomarkers, and may also have utility in predicting treatment response and resistance. Furthermore, uncovering new genomic drivers will help further research and advance clinical trials and facilitate personalised medicine approaches.

Next-generation RNA sequencing and oligonucleotide arrays identified genomic alterations associated with NEPC. Using tissue sourced from benign prostate, prostate adenocarcinoma, and NEPC, a significant overexpression and gene amplification of the protooncogenes MYCN and its stabilising cofactor Aurora kinase A (AURKA) were discovered in 40% of NEPC and 5% of prostate adenocarcinomas [71]. MYCN amplifications are also found in around 25% of neuroblastomas, correlating with poor prognosis and representing a well-characterised and important clinical marker [72]. As such, studies have looked at MYCN as a driver of the NE transdifferentiation process. One study reported that aberrant overexpression of the N-myc protein in accordance with the PTEN loss or AKT1 overexpression was sufficient to induce tumours with phenotypic and molecular NE features from primary human prostate epithelial cells [73]. Similarly, it has been shown in pre-clinical models that N-myc overexpression results in poorly differentiated, invasive PCa with molecular similarities to NEPC, including loss of AR signalling and induction of PRC2 signalling through EZH2 [74]. Furthermore, MYCN can induce the repression of microRNA miR-421 to ultimately activate ATM serine/threonine kinase and its signalling pathway that contributes to NE transdifferentiation [75].

Targeting MYCN and its associated factors has been extensively researched [76]. A number of AURKA inhibitors have been tested in clinical trials for the treatment of various cancers, such as barasertib, alisertib, danusertib, AT9283, PF-03814735, and AMG 900 [77]. Notably, a phase II trial was completed to evaluate MLN8237/Alisertib to treat NE mCRPC (NCT01799278) [78]. The trial was unable to meet its primary endpoint; however, a subset of patients achieved significant clinical benefits from the single-agent treatment. Similarly, in other trials involving AURKA inhibitors, none have progressed into the clinic as a standard-of-care due to issues with toxicity, efficacy in solid tumours, and pharmacokinetics. Since this is the case, singular treatment indirectly targeting MYCN via AURKA may not be a viable option for NEPC treatment; however, an in vivo neuroblastoma study has shown a synergistic inhibition of tumour growth and MYCN expression with a BRD4 inhibitor (I-BET151) and alisertib [79]. It has been proven that BRD4 inhibition can hinder NE-associated lineage plasticity [80], and thus this combination treatment appears to be a promising approach for the treatment of NEPC. Other avenues of MYCN inhibition that should be explored include indirect suppression by targeting MYCN transcription via CDK7 inhibitors, by destabilising MYCN (e.g., via MYCN-targeting PROTACS), by inhibiting MYCN transcriptional activity (e.g., small-molecule inhibitors like KJ Pyr-9) or by direct targeting [76]. One study has shown successful direct targeting and downregulation of MYCN using a DNA alkylating molecule called MYCN-A3 to quell neuroblastoma growth [81]. It should be noted that SOX11, an upstream gene and regulator of MYCN-induced NE transdifferentiation and proliferation, can be targeted and is a candidate as a novel therapeutic strategy for NEPC [82]. Since MYCN is a pioneer transcription factor, playing a large role in NE transdifferentiation and aggressiveness of the disease [71], successful targeting would most likely reap great benefits for patients and should be explored further.

One Cut Homeobox 2 (ONECUT2) has also been identified as a driver of NEPC. The ONECUT2 gene encodes a TF which has been demonstrated to play a role in the development and progression of NEPC [83]. ONECUT2, however, plays a dominant role in the progression to NEPC and has been shown to be frequently overexpressed in NEPC, promoting the expression of genes involved in NE transdifferentiation and growth, including the following: Achaete-scute homolog 1 (ASCL1), a TF that has been recently shown to activate neuronal stem cell-like lineage programming through chromatin remodelling, contributing to NEPC development [84]. In addition to this, ONECUT2 was found to be associated with increased resistance to ADT in PCa patients, and to contribute to this resistance by promoting the survival and growth of NEPC xenograft models in the absence of AR signalling. ONECUT2 has been shown to bind to the FOXA1 promoter and inhibit its expression [85]. Furthermore, recent studies have shown that targeting ONECUT2 could be a promising therapeutic strategy for NEPC. In preclinical studies, inhibiting ONECUT2 expression or its activity has been demonstrated to inhibit the growth and survival of NEPC cells. Indeed, a small-molecule inhibitor of ONECUT2, CSRM617, exhibited direct binding to the protein and successful tumour growth inhibition in vivo [85]. This suggests that targeting ONECUT2 may be a viable approach for treating this aggressive subtype of PCa; however, further drug optimisation and development are required [85].

During the discovery of ONECUT2 as a master regulator of NEPC, it should also be noted that TFs myelin transcription factor 1 (MYT1), SIX Homeobox 2 (SIX2), and myelin transcription factor 1-like (MYT1L) demonstrated functional neuronal development roles in NE tumours, while zinc finger protein 711 (ZNF711) showed expression correlations with NE tumour types [83]. Thus, these genes also play a role in the NE transdifferentiation but have not been studied further to date, requiring a deeper investigation.

ASCL1 is a TF known to activate neuronal stem cell-like lineage programming by chromatin remodelling. A study observed that enzalutamide treatment in castration-resistant models altered chromatin accessibility, and especially increased accessibility at promoter regions compared to other regions of the DNA. Moreover, consensus binding sequences for ASCL1 and other neuronal lineage TFs were enriched [84]. Mechanistically, ASCL1 is regulated by SOX2, required for EZH2-mediated cistrome reprogramming, and is hypothesised to be in a positive feedback loop with CREB signalling to resist ferroptosis, enhancing NE transdifferentiation [84,86,87,88,89]. Furthermore, one study has shown ASLC1 overexpression facilitated the creation of de novo regulatory elements associated with neuronal differentiation [90]. The overexpression of ASCL1 in vitro demonstrated that the TF induced stem and neuronal programs, increasing NE marker expression, and suggested that ASCL1 alone could induce cell plasticity and the NE phenotype [84,91]. Conversely, the loss of this TF initiated a lineage switch back to a luminal phenotype [84]. Interestingly, however, the concomitant loss of ASCL1 in vivo in one study did not stop NE small cell tumour formation, suggesting a compensatory mechanism by other NE drivers [91]; however, a similar in vivo study showed moderate tumour control by ASCL1 knockout [92]. In vivo experiments confirmed that ASCL1-driven NEPC requires a certain genomic landscape such as Rb1 loss and initial KRT8 expression in luminal epithelial cells that is gradually switched off during the NE transdifferentiation process [92].

A BET inhibitor, JQ-1, has been used in multiple studies for the treatment of Cushing’s disease and SCLC with the main implication of treatment being ASCL1 downregulation, suppression of its activity and downstream targets [93,94]; therefore, this could be a therapeutic avenue for the exploration of NEPC treatment. ASLC1 has a known downstream target in DLL-3, and is known to be important in NE tumours. DLL-3 demonstrated differential expression in NE PCa tumours compared to adenocarcinoma, correlated with NE markers, RB1 loss and aggressive clinical features. In vitro and in vivo inhibition of DLL-3 by Rovalpituzumab tesirine in NEPC models showed growth inhibition [95]. Indeed, phase III trials for SCLC involving the therapeutic, Rovalpituzumab (NCT03061812), a DLL-3-targeting agent, showed promise, however, was less efficacious than the standard of care treatment [96]. There may still be scope, however, for this method of therapy to be successful in NEPC: a phase Ib trial was recently completed using DLL-3-engager tarlatamab, showing “manageable safety with encouraging anti-tumor activity in DLL3 expressing NEPC” [97]. The results of subsequent trials will be important to gauge whether this method of inhibition is efficacious in NEPC. The BAP1 axis has been demonstrated to be upstream of ASCL1 and the inhibition of this showed significant ASCL1 downregulation. Specifically, a study was conducted in SCLC models, targeting the BAP1/ASXL3/BRD4 epigenetic axis. Treatment with iBAP-II repressed neuroendocrine lineage-specific ASCL1/MYCL/E2F signalling, decreasing cell viability and tumour growth [98]. Indeed, another study looking to treat neuroblastoma, demonstrated that BAP1 inhibition also destabilised MYCN and thus inhibited MYCN-mediated oncogenic signalling [99]. With both of these cancers having similar characteristics and activated signalling pathways as NEPC, BAP1 inhibition could be a promising avenue to explore for the treatment of this disease [98,99].

The master neural TF, POU domain, class 3, transcription factor 2 (BRN2) has been implicated in NEPC progression and has been shown to be negatively regulated by AR. When AR expression is lost, BRN2 can regulate the expression of SOX2 and cause lineage reprogramming to NEPC [17,50]. BRN4 (encoded by the POU3F4 gene) was also recently identified as a novel driver of the NE transdifferentiation process in the context of CRPC. Much like BRN2, BRN4 was found to be amplified and overexpressed in NEPC samples compared to CRPC, and was capable of driving progression to NEPC in a synergistic manner with BRN2, i.e., through SOX2 regulation and the initiation of a pro-neural programme [100]. Furthermore, BRN2/4 mRNA was discovered to be actively released into extracellular vesicles (EVs) during the NE transdifferentiation process, and it was also discovered that this effect was augmented by treatment with enzalutamide. The authors hypothesised that these EVs containing BRN2/4 mRNA could cause further NEPC induction through horizontal transfer to neighbouring cells. Interestingly, the release of BRN2/4 mRNA into EVs make these potential non-invasive biomarkers for predicting and diagnosing NEPC. The Zoubeidi lab recently published a study discovering the first-in-field BRN2 inhibitor for the treatment of NEPC, B18-94, that disrupts BRN2 interaction with DNA [101,102]. This inhibition showed downregulation of NE markers and decreased cell viability in NEPC models but no effect in adenocarcinoma, demonstrating its specificity to BRN2-overexpressing tumour models. Furthermore, tumour growth in vivo was decreased, with no toxicity observed, making this treatment a very attractive option to take into further studies [101]. Currently, there are no known BRN4 inhibitors.

Prospero homeobox protein 1 (PROX1) was identified in transcriptomic data to be upregulated in NEPC compared to CRPC [83], as well as showing increased expression in rectal NE tumours. Its increased expression correlates with cellular plasticity and NE transdifferentiation [13,103]. Mechanistically, PROX1 downregulates Notch1 signalling to induce neurogenesis [104]. A study by Wu et al. confirmed PROX1 upregulation in NEPC clinical samples, PDX models, transgenic mice, and cell models due to TP53/RB1 loss, PROX1 gene amplifications, and hypoxia. Further, they showed an inverse relationship between AR and PROX1 expression, and were able to reverse NE transdifferentiation and cellular plasticity with PROX1 knockdown in vitro and in vivo to restore enzalutamide sensitivity [105]. Raj et al. further demonstrated that TP53 and RB1 loss is critical for PROX1-induced NE transdifferentiation [106]. Currently, literature searches yield no known PROX1 inhibitor. However, the microRNA miR-140-5p has been shown to target PROX1 to regulate the proliferation and differentiation of neural stem cells [107]; thus, synthesising a molecule mimicking miR-140-5p to target PROX1 for the treatment of NEPC could be an interesting approach. Furthermore, researchers have used circular decoy DNA elements, CIRC and PSCIRC, that contain the binding sites of PROX1 and its interacting transcription factor, SOX18, to block PROX1 chromatin interactions [108]. These decoy molecules demonstrated stability in vitro and thus have potential as a PROX1-targeted treatment. Finally, targeting the PI3K/AKT pathway via the PI3K inhibitor, LY294002, was able to reduce PROX1 protein expression in in vitro follicular thyroid carcinoma cell models [109]. While these PROX1-targeting therapies are in early stages of development, repurposing them and developing them further could yield promising results for NEPC treatment.

The TF, Forkhead Box A2 (FOXA2) was discovered to have high expression in NEPC cell lines and PDX models compared to being absent or low-expressed in prostate adenocarcinoma, with its expression promoted by ADT [110,111]. It is suggested that FOXA2 should be considered a lineage survival factor in NEPC by promoting an NE phenotype whilst suppressing alternative lineage programs (e.g., luminal/epithelial programs). FOXA2 also has shown to have an alternate transcriptional program in NEPC compared to prostate adenocarcinoma, involving genes associated with neurogenesis, neurotransmission, and neuropeptide signalling, facilitated by cooperation with HIF-1α [112]. For example, downstream activation of the Kit pathway facilitates NE cell-specific communication. Furthermore, loss of FOXA2 in NEPC cells downregulates NE markers and upregulates epithelial markers, suggesting its role in the transdifferentiation process [110]. In a similar fashion, FOXB2 and FOXC2 have been shown to promote NEPC through the activation of Wnt signalling and EMT/CSC, respectively [113,114,115]. There are no direct inhibitors of FOXA2; however, targeting upstream factors to inhibit FOXA2 activity may be a viable option. One study showed that the antagonistic targeting of EGFR was able to inhibit FOXA2 [116]. A multitude of EGFR inhibitors are commercially available for cancer treatment [117], and should thus be considered for the treatment of NEPC. Linc00261, a long noncoding RNA has also shown to be upstream of FOXA2 and drive its expression [118]. Although no inhibitors for this molecule have been discovered, this may be more easily targetable compared to FOXA2 directly, and thus, the discovery and synthesis of small molecule inhibitors for Linc00261 should be explored. Many endogenous factors are known to inhibit FOXA2 expression, namely an array of microRNAs (e.g., miR-200, miR-141-3p), HDAC3, and PGC-1β [118]. Potential mimicry molecules, especially of the microRNAs, may be a fruitful avenue to explore the treatment of FOXA2-driven NEPC.

Neuronal differentiation 1 (NEUROD1) is a known regulator of nervous system development, shown to reprogram chromatin and TF landscapes to induce the neural program [119]. The expression of this TF is correlated to a high CHGA expression and mechanistically collaborates with MYCN to initiate NE oncogenesis [13,120]. One study, however, showed that expression with ASCL1 is mutually exclusive, whereby NEPC PDX tumours either expressed one TF or the other. However, larger datasets are required to confirm this in patients [121]. However, this suggests a potential heterogeneity of NEPC and thus differing subtypes. Other studies have suggested that originally high ASCL1-expressing tumours evolved to create a subclone high in NEUROD1 and low in ASCL1 [13]. Like many of the factors discussed above, there are no known direct NEUROD1-targeting therapies; however, targeting its coactivator, BET, bromodomain proteins by BET inhibitors (JQ-1) has demonstrated efficacy in SCLC [122], suppressing NEUROD1 signalling and inhibiting growth in vitro.

Paternally expressed gene 10 (PEG10) is a retrotransposon-derived gene that plays a crucial role in cell proliferation and differentiation. Originally identified for its involvement in embryonic development, PEG10 has garnered attention for its significant role in various cancers, including PCa [123,124,125]. Studies have demonstrated that PEG10 is an AR-repressed gene that is upregulated in NEPC, promoting its growth and progression [126,127]. This upregulation is believed to be driven by complex genetic and epigenetic mechanisms that distinguish NEPC from other PCa subtypes. Whilst there are no commercially available therapies against PEG10, research has shown that transient knockdown approaches can cause growth suppression in AR-null PCa cells such as DU145 and PC3, and stable knockdown was shown to significantly reduce the growth of PC3 cells in a xenograft model [126]. Later research showed that the knockdown of PEG10 could reduce the in vitro proliferation of CRPC cells and cell lines with neuroendocrine-like features, and could attenuate tumour growth in a neuroendocrine-like in vivo xenograft model [127]. A recent breast cancer study demonstrated the in vitro and in vivo efficacy of a PEG10-targeting antisense oligonucleotide in combination with a CDK4/6 inhibitor, suppressing proliferation and EMT [128], and a similar approach could be taken in NEPC. Understanding the relationship between PEG10 and NEPC may provide insight into the molecular underpinnings of NEPC and may also open potential avenues for targeted therapies aimed at mitigating the effects of PEG10 overexpression in NEPC patients.

Several other TFs have been implicated in the progression to NEPC; these include, but are not limited to zinc finger and BTB domain containing 7A (ZBTB7A) [129], ZBTB46 [130], and DEK Proto-Oncogene (DEK) [131], all of which represent novel therapeutic targets. The first study demonstrated differential transcriptomes of ZBTB7A in NEPC vs. adenocarcinoma, while silencing of the transcription factor inhibited cell proliferation [129]. There are no inhibitors of ZBTB7A as of yet but it remains a targetable protein. Secondly, ZBTB46 directly upregulates Nerve Growth Factor (NGF) that interacts with cholinergic receptor muscarinic 4 to promote NE transdifferentiation [130]. The study demonstrated that the targeting of NGF reduces ADT resistance and neuroendocrine differentiation, but more research is needed to develop specific small-molecule inhibitors of ZBTB46 and evaluate their therapeutic potential in the context of NEPC. Finally, DEK exhibited a higher expression in NEPC clinical samples compared to adenocarcinoma, while the downregulation of it inhibited growth in vitro. No small-molecular inhibitors are available; however, DEK-targeting aptamers have shown some efficacy for the treatment of arthritis [132] and could therefore have repurposing potential for the treatment of NEPC.

### 2.4. Alterations in Androgen Receptor Interactors

It is well known within the field that AR is the main driver of PCa and NEPC arises as a resistance mechanism against the inhibitors of its pathway. However, the exact mechanisms of this resistance are still not fully known. Exploring the link between AR interactors and NE transdifferentiation is vital for understanding how alterations in AR signalling drive disease transformation and contribute to treatment resistance. A more comprehensive understating may also aid in identifying diagnostic and prognostic biomarkers related to AR activity, and facilitate the development of targeted therapies that address AR-related mechanisms.

To date, only one study has specifically looked at Homeobox (HOX) gene expression in its entirety in NEPC, and it was reported that HOX genes have an altered pattern of expression compared to other PCa types and that this is punctuated by the loss of HOXB13 expression [133,134]. The study showed, at both the transcript and protein levels, minimised HOXB13 expression in NEPC samples (mirrored in NEPC cell lines) [134]. The loss of expression was discovered to be due to the DNA hypermethylation of the HOXB13 gene. HOXB13 is the most extensively investigated HOX gene in PCa due to the discovery of germline mutations in familial PCa [135]. HOXB13 has been shown to act as a tumour suppressor in PCa through the inhibition of AR activity, thus functioning as an AR repressor and exerting a growth-inhibiting effect on PCa cells [136]. However, it may also act as coactivator of AR; recent studies have demonstrated that HOXB13 can enhance AR-V7 genomic binding activity, and that HOXB13 silencing in vitro and in vivo inhibited adeno-CRPC growth. More specifically, it was discovered that HOXB13 is required for and colocalises with AR-V7 binding to open chromatin across CRPC genomes. By advancing the binding of AR-V7 through a direct physical interaction, HOXB13 promotes the overexpression of target oncogenes, thereby promoting proliferation [137]. Therefore, the loss of HOXB13 in NEPC is congruent with the loss of AR expression and is suggested to be used as a marker to determine the phenotype of PCa in patients [138]. A potential mechanism of HOXB13 loss may be facilitated by EZH2, as it has been shown that EZH2 has a major role in the silencing of HOX gene expression during embryonic development [139], and this may also be the case in this scenario. HOXB13 loss is also associated with lipid accumulation in PCa cells (due to the loss of interaction with HDAC3), facilitating cell motility and metastasis [140].

Re-expressing HOXB13 might reverse NE transdifferentiation by subsequently re-expressing AR, though devising a strategy for this process appears complex. Similar to the studies that reintroduce TSGs, one colorectal cancer study reintroduced HOXB13 expression by an adenoviral vector system [141]. This study found that, like in PCa, HOXB13 loss was suggested to be an important event in colorectal cell transformation. The reintroduction of the gene by the vector system demonstrated a suppression of growth in vitro. Furthermore, the study discussed above mentioned that treatment with all-trans retinoic acid (ATRA) induced the re-expression of HOXB13 in cell models [134] and proved to be efficacious; therefore, this therapeutic avenue of reintroduction of the HOXB13 gene should be explored further to see whether this could be translated into the clinic.

FOXA1, a TF, is a key coregulator of AR, pioneering AR chromatin binding [142]. Various studies report differing findings on FOXA1 in NEPC: either it is lost/inhibited, or it has a different cistrome compared to adenocarcinoma. As previously mentioned, ONECUT2 has shown to bind FOXA1 promoter regions to inhibit its expression, while another report demonstrated that FOXA1 inhibits NE transdifferentiation and is therefore downregulated in NEPC [85,143,144,145]. After just one week of FOXA1 knockdown in LNCaP cells, morphological and phenotypic changes towards neuroendocrine cells were observed. It was discovered that FOXA1 loss induced IL-8 mediated MAPK/ERK activation and consequently NE transdifferentiation [143]. Conversely, another study showed that, in NEPC, FOXA1 reprogrammed its cistrome to NE-specific regulatory elements, driving the expression of NE lineage genes [142]. The study states that the FOXA1 promoter shed contacts with its canonical regulatory regions and looped to a distinct NEPC-restricted super enhancer, with both a distal super-enhancer and promoter co-bound by FOXA1 and ASCL1, suggesting autoregulation. The study also revealed that FOXA1 stimulated expression of FOXA2 and INSM1 [142]. There are no direct FOXA1 inhibitors; however, there are many indirect mechanisms of FOXA1 inhibition being explored in other cancers [118], but given the conflicting reports on its expression in NEPC, it is suggested that this should not be targetable in this disease.

### 2.5. Other Modulatory Factors

Several other factors involved in different processes have also been shown to influence the NE transdifferentiation process in PCa. Understanding these additional modulatory elements is crucial, as they offer new insights into the complex biology of NEPC and may reveal novel therapeutic targets. This section will explore these diverse modulatory factors, highlighting their potential roles and implications in NEPC pathogenesis.

Whilst there are encouraging studies with respect to EZH2 and its contribution to the NE transdifferentiation process, Li et al. made another important discovery that may shed some more light on how this process is driven. The researchers used a novel comparative alternative splicing detection tool to analyse alternative RNA splicing in two RNA-Seq datasets, namely The Beltran Cohort and the Vancouver Prostate Centre Cohort. Serine/Arginine Repetitive Matrix 4 (SRRM4) is an RNA-splicing factor that has been identified as a driver of the NE phenotype and as a potential target for novel therapies [146]. In the study, SRRM4 expression levels were found to be comparatively upregulated in NEPC with respect to adenocarcinoma samples in both datasets, and the researchers demonstrated a negative association with the expression of SRRM4 and the master transcriptional regulator RE1-silencing TF (REST), which silences neuronal genes in non-neuronal cells, preventing differentiation. The researchers suggested a potential mechanism whereby SRRM4 drives NE transdifferentiation by regulating REST expression. This was further evidenced by the ability of SRRM4 to alter cellular morphology and induce the expression of NEPC markers in adenocarcinoma cells. Currently, there are no known methods of inhibiting SRRM4 and/or upregulating REST expression, but this could be an interesting avenue to explore NEPC treatment.

CXCR2 is a chemokine receptor that is involved in various signalling pathways that promote tumour growth, angiogenesis, and metastasis [147]. Interestingly, tissue microarrays (TMAs) revealed CXCR2 to be specifically expressed on the surface of NE cells and not luminal cells, and further analysis revealed that the cell surface marker was enriched in high-grade PCa, with the highest expression seen in NEPC cells [148]. Functional studies revealed CXCR2 to be a driver of progression to NEPC. In this study, CXRC2 was functionally linked to the downregulation of AR, lineage plasticity and resistance to hormone therapy [148]. Importantly, the inhibition of CXCR2 with inhibitor navarixin was demonstrated to be effective in advanced mouse xenograft models in combination with enzalutamide, with the authors concluding that this combination may be effective in targeting both luminal and NE tumour cells, effectively addressing the heterogeneity seen in most cases of t-NEPC [148]. This could be an exciting approach to take into further studies.

One study revealed that protein kinase C (PKC)λ/ι is downregulated in de novo and NEPC. This causes serine production to be upregulated via a mechanism mediated by mTORC1/ATF4 [149]. Raised intracellular serine amino acid levels caused by this metabolic reprogramming results in increased S-adenosyl methionine levels, leading to the promotion of cell proliferation and epigenetic modifications that encourage the emergence of NEPC traits. This discovery presents a potential therapeutic target to prevent transition to NE transdifferentiation and therefore therapy resistance in PCa through the inhibition of mTORC1/ATF4 signalling.

Ret proto-oncogene or RET kinase (RET) is a receptor tyrosine kinase that is critical for the development and maintenance of various tissues, particularly the nervous system. RET also plays a role in mediating signal transduction pathways that regulate cell growth, proliferation, differentiation, and survival [150]. Activating point mutations in RET can lead to the hereditary cancer syndrome called multiple endocrine neoplasia type 2 (MEN 2) [151], and chromosomal rearrangements involving RET and other proteins can lead to the constitutive activation of RET, primarily being associated with malignancies such as papillary thyroid carcinoma [152]. A study using global phosphoproteomics comparing AR-independent and AR-dependent PCa cell lines to identify targetable kinases in NEPC discovered that RET kinase activity was enriched in AR-independent cell lines [153]. Further to this, clinical NEPC patient samples and patient-derived xenografts showed an upregulated RET transcript and pathway activity, and the genetic knockdown or pharmacologic inhibition of RET kinase significantly reduced tumour growth and cell viability in multiple NEPC models, including an NCI-H660 xenograft model [153]. The study found that targeted RET pathway inhibitors (AD80, LOXO-292, and BLU-667) were more effective in inducing cell death compared to the currently approved RET inhibitor therapies like cabozantinib and vandetanib. More recently, cancer cells exhibiting high small-cell neuroendocrine phenotype scores have demonstrated a strong dependency on RET kinase activity, with a significant correlation between RET and ZBTB7A dependencies [129]. Currently, selpercatinib and pralsetinib are the only FDA-approved RET kinase inhibitors and are used to treat diseases such as NSCLC [154,155]. These drugs may be repurposed in the future for the treatment of NEPC with high RET expression. A currently recruiting phase II clinical trial is investigating the multi-kinase inhibitor ESK981 in patients with select solid tumours (NCT05988918), that includes a number of neuroendocrine tumours inclusive of NEPC [156]. ESK981 has the ability to inhibit RET alongside other kinases such as VEGF, FGFR1, and TIE2. The results from this trial will prove extremely useful to determine the utility of this mode of inhibition.

The role of long non-coding RNAs (lncRNAs) in the NE transdifferentiation process is a relatively new focus of research. The lncRNA H19 was identified by a study to be upregulated in NEPC and drive changes in stem cell and NE genes when overexpressed in vitro [157]. The opposite was observed in a stable knockdown system of the lncRNA, causing a regression of the NE phenotype and a resensitisation of cells to enzalutamide. Particularly, H19 activity in PCa is facilitated by RB1/TP53 loss, and a biphasic increase in expression is observed firstly after castration and secondly during the NE transdifferentiation process. It was also discovered that SOX2 regulates the expression of H19. Mechanistically H19 induces epigenetic changes such as histone methylation by binding to the PRC2 complex, thus driving EZH2 activity. This evidence suggests H19 to be a diagnostic and predictive biomarker of NEPC. A H19-targeted therapy, a DNA plasmid H19-DTA, was assessed in clinical trials for ovarian and bladder cancers, exhibiting good safety profiles and efficacy against tumour growth [158]; therefore, there is potential to use this therapeutic approach for NEPC additionally.

Notably, other lncRNAs have been identified to be dysregulated in NEPC but not fully investigated for whether they play a role in the NE transdifferentiation process, offering the scope for further examination [159,160].

## 3. Discussion and Conclusions

**Figure 3:**
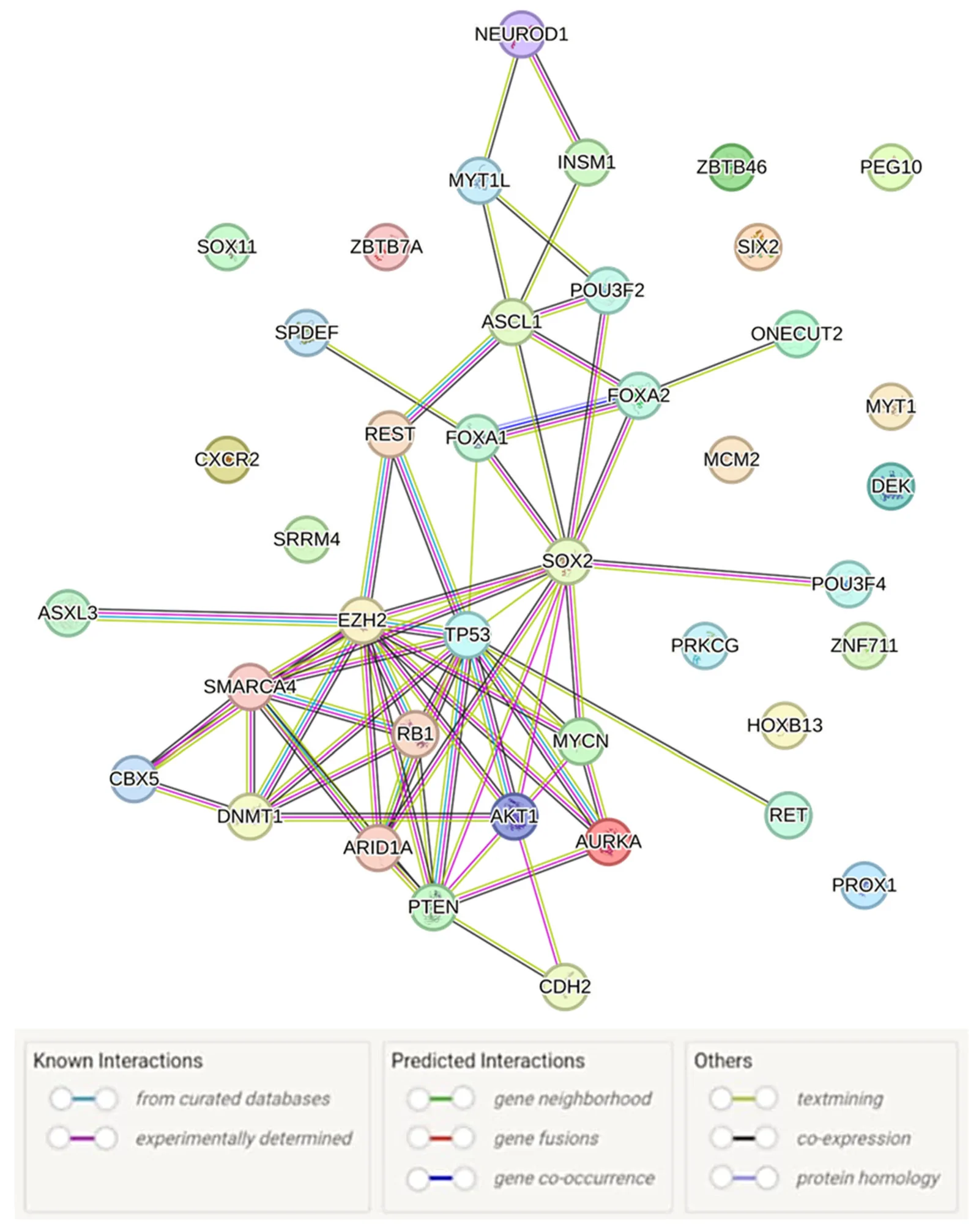
Protein–protein interaction network of driver NE transdifferentiation genes. Confirmatory analysis of the mentioned genes shows interconnectedness to drive NE transdifferentiation. Network analysis using STRING, with the interaction score set to a confidence threshold of 0.700. Core proteins such as TP53, RB1, MYCN, and EZH2 are central to the network with greater confidence scores, highlighting their significance in NEPC transdifferentiation. Line thickness indicates the strength of the data supporting the interactions. Gene names (not encoded protein names) used: ARID1A and SMARCA4 used as main SWI/SNF signalling genes. The confidence scores are computed by STRING using evidence channels (textmining, experiments, databases, co-expression, neighbourhood, gene fusion, and co-occurrences). The colours correspond to different “evidence channels” in STRING, each representing a distinct type of data or method used to infer protein associations: multiple colours on a single line suggest multiple types of evidence for that interaction [161].

NEPC is a complex disease, with increasing patient numbers due to the increased use of AR-targeted therapies, and is the subject of a growing body of scientific and medical research. The NE transdifferentiation process is not yet fully understood; however, genetic, epigenetic, and other factors described above contribute to lineage plasticity, cell cycle dysregulation, and resistance to hormone therapy (Table 1). These changes facilitate a phenotypic and lineage switch from an adenocarcinoma to a neuroendocrine phenotype. The drivers of NEPC are interconnected, often regulating each other’s expression and activity, and involve multiple biological pathways. From this, it can be deduced that the NE transdifferentiation process is multifaceted, requiring a plethora of changes in the genome and epigenome; thus, not one singular gene is responsible for these alterations. Rather, a network of genes and epigenetic factors are responsible, all with implicating effects on each other. This is confirmed by STRING network analysis (Figure 3) [161], whereby the protein–protein interaction network of the aforementioned factors is crucial for NE transdifferentiation. Focusing on central genes with high confidence scores and multiple interactions, TP53, MYCN, EZH2, and RB1 emerge as key players in NE transdifferentiation. Functional enrichment of the network shows GO biological processes for neurogenesis and negative regulation of cell differentiation, and KEGG pathways of PCa. Therefore, for the field of treatment for NEPC to progress, combination therapy targeting multiple factors and key players should be considered the most favourable approach. It can be assumed that by targeting solely one of the genetic factors discussed above, compensatory mechanisms within the tumour may occur and therefore result in resistance.

From the findings of this review, it can be suggested that combination therapies should focus on the following: gene therapy for the reintroduction of TSGs, BET inhibitors, and/or EZH2 inhibitors. The reintroduction of TSGs could be vital to reversing the neuroendocrine transdifferentiation process back to an adenocarcinoma phenotype. Moreover, since these genes play key roles in processes such as the cell cycle, survival, growth, metabolism, and DNA damage responses, and have been shown to act as a “catalyst” for NEPC development, the re-expression of these tumour suppressors could be pivotal to NEPC treatment. Viral vector systems and other mechanisms for wild-type TSGs to regain expression should be a priority when developing novel treatments for this lethal disease, where the results of the ongoing clinical trials involving these therapies will give insight into the efficacy of the treatment.

Considering that BET inhibitors have demonstrated the inhibition and downregulation of the TFs, MYCN, ASCL1, and NEUROD1, three factors that have key roles in the NE transdifferentiation process, it is strongly suggested that this therapeutic avenue should be further explored for the treatment of NEPC. Extensive existing research and development of BET inhibitors [162] makes this approach even more attractive, whereby knowledge from previous studies can facilitate the determination of specific BET inhibitors that can be repurposed for NEPC treatment, leading to a more streamlined procedure.

Epigenetic targeting, for example, tazemetostat against EZH2, looks to be a promising and exciting field. As with many cancers, more personalised and tailored treatments tend to focus on the genetic factors at play and only recently have epigenetic factors been recognised. Considering that EZH2 activity has been demonstrated to play a large role in the progression and phenotypic changes associated with NEPC, and since tazemetostat is currently in clinical trials for the treatment of mCRPC with inclusion criteria for NE prostate tumours, the results in the later end of this year will give insight into the efficacy of this treatment option.

It is also important to note the similarities of NEPC with other, more researched cancers such as SCLC and neuroblastoma. As a scientific community, it should be important to recognise the similarities and use this knowledge to apply successful treatments from one cancer to another. Genetically, many neuronal markers are upregulated in these cancers such as ASCL1 and MYCN, and the downregulation of factors such as TP53 and RB1. It should also, therefore, be considered that the immunological landscape of these cancers may be the same as that of the expression of checkpoint genes PD-L1 and CTLA-4 [163]. Immunotherapy for cancers is a relatively new approach that should also be considered for NEPC but requires further extensive research. A phase II clinical trial for NEPC determining the success of PD-L1 inhibition showed variable results, whereby the presence of microsatellite instability increased treatment efficacy [164]. Interestingly, the FDA has chosen to fast-track an innate immune activator, BXCL701, in combination with pembrolizumab in mCRPC patients that present with an NE phenotype due to its success in phase IIa clinical trials. The treatment demonstrated encouraging anti-tumour activity with durability of response in late-line, refractory small cell NE mCRPC [165].

It should be noted that researchers have been attempting to treat the broad spectrum of NE tumours (more commonly gastrointestinal or pancreatic tumours) using lutetium-labelled peptides [166]. This is a form of endo-radiotherapy that combines a radionuclide with a peptide that binds specifically to somatostatin receptors which are expressed at a high concentration on tumour cells [167]. FDA approval was granted to treat gastroenteropancreatic NE tumours, while several other clinical trials are also underway to treat a wide range of NE tumours [168,169,170]. Indeed, one phase II trial has specifically focused on metastatic NEPC tumours (NCT05691465), with the completion date expected to be in late 2025 [171]. Building on the efficacy seen in other NE neoplasms, this trial may reveal a new therapeutic approach and critical insights for targeted treatment.

It is essential to recognise tyrosine kinase inhibitors as a viable option for the treatment of NEPC, although they may not directly be targeting the drivers of transdifferentiation. A few clinical studies are currently in place, such as NCT01799278 and NCT05988918, as previously mentioned [78,156], targeting RET kinase and AURKA, respectively. The final trial, NCT04848337, using combination therapy of pembrolizumab, a PD-1 inhibitor and lenvatinib, a VEGF inhibitor [172], is currently recruiting patients with advanced/metastatic NEPC. This interesting combination treatment may prove effective in treating the disease.

Many of the markers mentioned that drive the NE phenotype have been discovered relatively recently, and thus require more investigation into their mechanism of action and whether they can provide therapeutic targets. PROX1 seems to play an early role in the NE transdifferentiation process but is yet to be fully explored and has limited known targeted therapies. The mechanistic investigation and targeting of PROX1 could yield very fruitful results for the treatment of NEPC, since if cells are halted at the early stage of the transdifferentiation process, the reversal and treatment of the disease may be less onerous for patients. The RNA splicing factor SRRM4 and its role in NEPC pose as a different approach compared to most of the other factors discussed. The mechanism requires further investigation, but the results could lead to a novel therapeutic target of NEPC.

Ideally, combination therapy to treat NEPC will reactivate AR signalling, mediating the luminal epithelial phenotype, making the cancer sensitive to androgens and thus androgen therapy. It can be advised that once the cancer is again AR-sensitive, a combination of AR inhibitors [3] and therapy to halt the retransdifferentiation process should be given.

Such an expansion of knowledge on drivers of NEPC and potential therapeutic avenues allows researchers to understand the mechanisms of the cancer and how to treat it best. This will, hopefully, lead to the development of specific treatments for NEPC, extending the life of patients and improving their quality of life.

**Table 1:** Key drivers in neuroendocrine prostate cancer (NEPC) development and potential therapeutic strategies. This table summarises the critical molecular drivers implicated in NEPC development, their roles in disease progression, and the corresponding potential therapeutic interventions.

Driver	Role in NEPC Development	Potential Therapy	References
AKT1	Overexpressed with N-myc.	Capivasertib	[11]
ASCL1	Activation of neuronal stem cell-like lineage programming through chromatin remodelling. ONECUT2 target gene. Required for EZH2 activity.	Upstream inhibition of BET = JQ-1 or BAP1 inhibition = IBAP-II. Downstream inhibition of DLL-3 = Rovalpitzumab, Tarlatamab	[84,90,93,99]
AURKA	Amplified and overexpressed, stabilising cofactor for N-myc.	Borasertib, Alisertib, Donusertib, AT9283, PF-0381475	[71,76,80,82]
BRN2	Upregulated. Cell lineage reprogramming through coregulation of SOX2 with BRN4.	B18-94	[17,100,101,102]
BRN4	Amplified and overexpressed. Cell lineage reprogramming through coregulation of SOX2 with BRN2. Released into EVs upon transdifferentiation and enzalutamide treatment. Horizontal transfer of EVs containing BRN2/4 mRNA to neighbouring cells.	-	[100]
CXCR2	Drives loss of AR expression, lineage plasticity, and hormonal resistance.	Navarixin	[148]
DEK	Elevated expression, facilitates transition to NEPC.	DEK-aptamers	[131,132]
Epigenetic alterations	Dysregulation of SWI/SNF, upregulation of DNMT1, hypermethylation of SPDEF and ASXL3 and hypomethylation of INSM1 and CDH2. These epigenetic changes contribute to the NE phenotype and have potential as markers for detection.	-	[67,68,69]
EZH2	Upregulated due to TP53 and RB1-loss. Fosters stem cell-like epigenetic environment, permitting lineage plasticity, progression to NEPC and drug resistance.	DZNep, Tazemetastat, inhibition of regulators: miRNA, MiR-101, miR-26a or lncRNA, MALAT1, TUG1	[30,56,57,60,61,62,63,74]
FOXA1	Dysregulated thus as an AR coregulator, facilitates AR signalling dysregulation.	-	
FOXA2	Highly expression alters transcriptomic landscape. Induces Kit expression to facilitate NE cellular communication.	Upstream inhibition of EGFR, Linc00261. Mimicry of molecules miRNAs, HDAC3 or PGC-1B	[110,111,116,117,118]
H19	LncRNA overexpressed in NEPC, shown to drive NE gene and stem cell gene expression. Binds PRC2 in implement epigenetic changes.	H19-DTA	[157,158]
HP1α	Expression levels increase as an early event and rise steadily during the transdifferentiation process to NEPC via a mechanism that involves the repression on the promotors of AR and REST.	Inhibition of phosphorylater: Nuclear Dbf2 related kinase	[65,66]
HOXB13	Loss in NEPC. Regulator of AR.	All-trans retoanoic acid, Gene therapy to reintroduce wild-type expression	[133,134,141]
MYCN	Overexpression and amplified alongside stabilising cofactor AURKA. N-myc protein overexpression results in poorly differentiated, invasive disease with loss of AR signalling and induction of PRC2 signalling.	Direct targeting: MYCN-A3, indirect targeting: CDK-7i, SOX11i, PROTACS, BRD4i e.g., I-BET151, KJ Pyr-9	[71,73,74,76,77,79,80,81,82]
MYT1	Regulator of NE tumour genes, coexpressed and regulated by ONECUT2, increased with hypoxia.	-	[71]
MYT1L	Upregulated, can reprogram and maintain cells toward NE phenotype.	-	[83,173]
NEUROD1	Early regulator of NE development, collaborating with MYCN to drive NE phenotype.	Inhibition of BET coactivator = JQ-1	[119,120,121]
ONECUT2	Overexpressed, induces ASCL1 to cause lineage reprogramming. Associated with increased resistance to ADT. Promotes survival and growth of NEPC cells.	CSRM617	[83,85]
PEG10	Derepressed during adaptive response to antiandrogen therapy and highly upregulated.	PEG10-ASO	[126,127,128]
Protein kinase C (PKC)λ/ι	Downregulated, supports transition to NEPC via mTORC/ATF4 signalling and S-adenosyl methionine abundance.	Inhibition of regulating signalling axis: mTORC1/ATF4	[149]
PROX1	Facilitates NE transdifferentiation process, upregulated. TP53/RB1 loss is critical.	Mimicry of regulator miR-140, Decoy molecules to prevent chromatin interactions, PI3Ki e.g., LY294002	[105,106,107,108,109]
PTEN	Frequently alteratered/lost. Promotes RB1 loss, facilitating lineage plasticity and metastasis. Alongside AKT1 overexpression of TP53 loss, induced NE molecular features and insensitivity to anti-androgens.	Gene therapy to reintroduce wild-type expression, downstream inhibition of AKT = Capivasertib	[27,30,37,38,39,41,42,73]
RB1	Retinoblastoma protein (Rb) loss and RB1 allelic loss promotes lineage plasticity and metastasis, which is initiated by PTEN mutation. No longer represses the expression of epigenetic reprogramming factors, such as SOX2 and EZH2.	Gene therapy to reintroduce wild-type expression, downstream inhibition of cell cycle checkpoint proteins or PARP	[27,28,30,36,43,44,45,46]
REST	Downregulated by SRRM4 and HP1α during ADT facilitating neuroendocrine transformation.	-	[65,146]
RET	Upregulated receptor tyrosine kinase supports NE transdifferentiation.	AD80, LOXO-292, BLU-667, cabozantinib, vandetanib, Selpercatinib, ESK981 and Pralsetinib	[129,153,154,155,156]
SIX2	Demonstrated functional neuronal development role in NE tumours.		
SOX2	Regulated by BRN2/BRN4, causes cell lineage reprogramming to a neuroendocrine phenotype. Suppressed by RB1 and TP53 in adenocarcinoma cells. Promotes lineage plasticity and enzalutamide resistance with loss of TP53 and RB1. Regulates ASCL1.	Upstream inhibition of LIN28 e.g., TPEN, LI71. Downstream inhibition of LSD1 e.g., TCP, ORY-1001, GSK-2879552, IMG-7289	[17,29,50,51,52,53,100]
SOX11	Upstream and regulator of MYCN to induce NE transdifferentiation.	-	[82]
SRRM4	Upregulated in NEPC, negatively associated with REST. May regulate REST expression and drive neuroendocrine transformation.	-	[146]
TP53	High mutation frequency/loss in NEPC. Loss confers resistance to antiandrogen therapy. Necessary alongside RB1 to repress the expression of SOX2 and EZH2.	Gene therapy to reintroduce wild-type expression, inhibition of negative regulators MDM2/MDMX	[27,28,30,31,32,33,34,35,47]
ZBTB46	Regulates nerve growth factor, inducing NE transdifferentiation.	-	
ZBTB7A	Has a distinct network pattern in NEPC vs. adenocarcinoma, strongly associated with cell cycle progression and regulation of apoptosis.	-	[129]
ZNF711	Overexpressed to promote NE transdifferentiation.	-	[83,174]

## Data Availability

No new data were created or analyzed in this study. Data sharing is not applicable to this article.
