# Correction: Maylin et al. Therapeutic Exploitation of Neuroendocrine Transdifferentiation Drivers in Prostate Cancer. *Cells* 2024, *13*, 1999

**DOI:** 10.3390/cells15181636

**Published:** 2026-09-10

**Authors:** Zoe R. Maylin, Christopher Smith, Adam Classen, Mohammad Asim, Hardev Pandha, Yuzhuo Wang

**Affiliations:** 1Vancouver Prostate Centre, Department of Urological Sciences, University of British Columbia, Vancouver, BC V6H 3Z6, Canada; 2Department of Experimental Therapeutics, BC Cancer Agency, Vancouver, BC V5Z 4E6, Canada; 3Targeted Cancer Therapy, Department of Clinical and Experimental Medicine, Faculty of Health and Medical Sciences, University of Surrey, Guildford GU2 7WG, UK

## Unreliable Citations

In the original publication [1], some of the references cited have had recent scrutiny by the academic community.

As such, Tabrizian et al. (originally reference 86) has been removed from the manuscript while other references have been added in exchange for this citation.

In exchange for Tabrizan et al., additional references: Yang et al. (2025) [2], Li et al. (2024) [3], and Ding (2025) [4] were added and are now reference numbers 86, 88 and 89 in the new edited manuscript. The citations have now been inserted in 2.3 Genomic Neuroendocrine Drivers Section, Paragraph 6 and should read:

Corrected paragraph: Mechanistically, ASCL1 is regulated by SOX2, required for EZH2-mediated cistrome reprogramming, and is hypothesised to be in a positive feedback loop with CREB signalling to resist ferroptosis, enhancing NE transdifferentiation.

In various other places throughout the manuscript, additional citations have been added to supplement other references in order to support statements made in the text. Additional citations are named below:

Zheng et al. (2024) [5] was not cited in the original manuscript but has now been added, supplementing statements made by questioned reference 57, Zhang et al. The citation has now been inserted in 2.2 Epigenetic Factors Section, Paragraph 3. Zheng et al. and Zhang et al. are now reference numbers 57 and 58.

Børretzen et al. (2019) [6] was not cited in the original manuscript but has now been added, supplementing statements made by questioned reference 110, Paranjape et al. The citation has now been inserted in 2.3 Genomic Neuroendocrine Drivers Section, Paragraph 10. Paranjape et al. and Børretzen et al. are now reference numbers 115 and 113.

Fu et al. (2016) [7] and Potter et al. (2012) [8] were not cited in the original manuscript but has now been added, supplementing statements made by questioned reference 138, Kim et al. The citations have now been inserted in 2.4 Alterations in Androgen Receptor Interactors Section, Paragraph 5. Kim et al., Fu et al. and Potter et al. are now reference numbers 143, 144 and 145.

Duan et al. (2025) [9] was not cited in the original manuscript but has now been added, supplementing statements made by questioned reference 103, Ding et al. The citation has now been inserted in 2.3 Genomic Neuroendocrine Drivers Section, Paragraph 9. Ding et al. and Duan et al. are now reference numbers 107 and 103.

## References

With this correction, the order of some references has been adjusted accordingly.

The authors state that the scientific conclusions are unaffected. This correction was approved by the Academic Editor. The original publication has also been updated.
